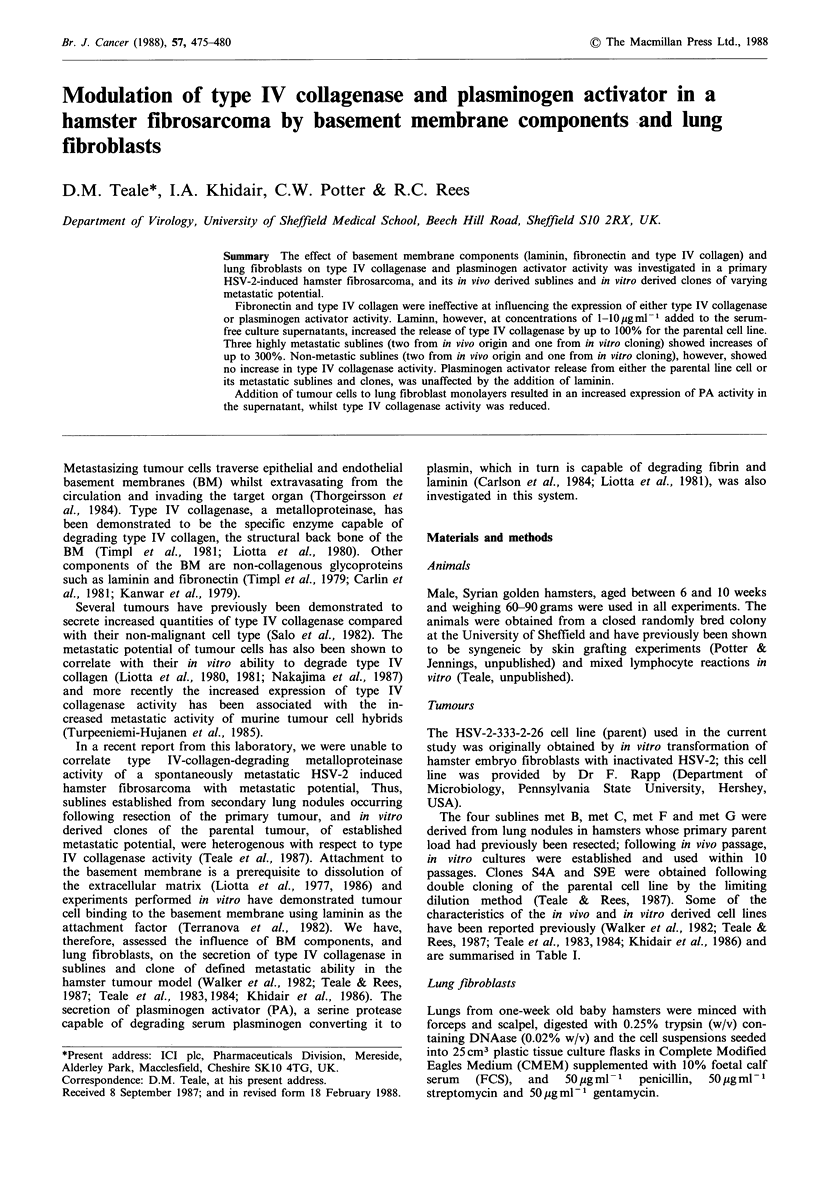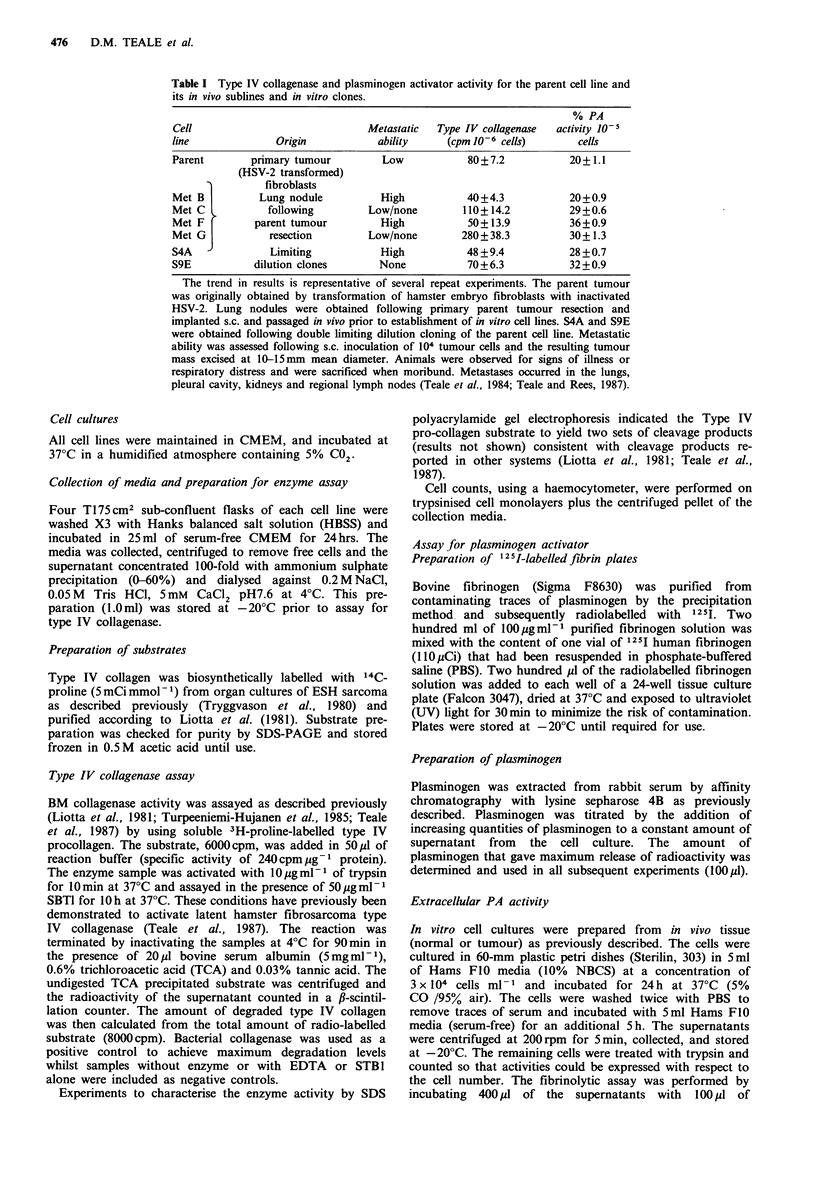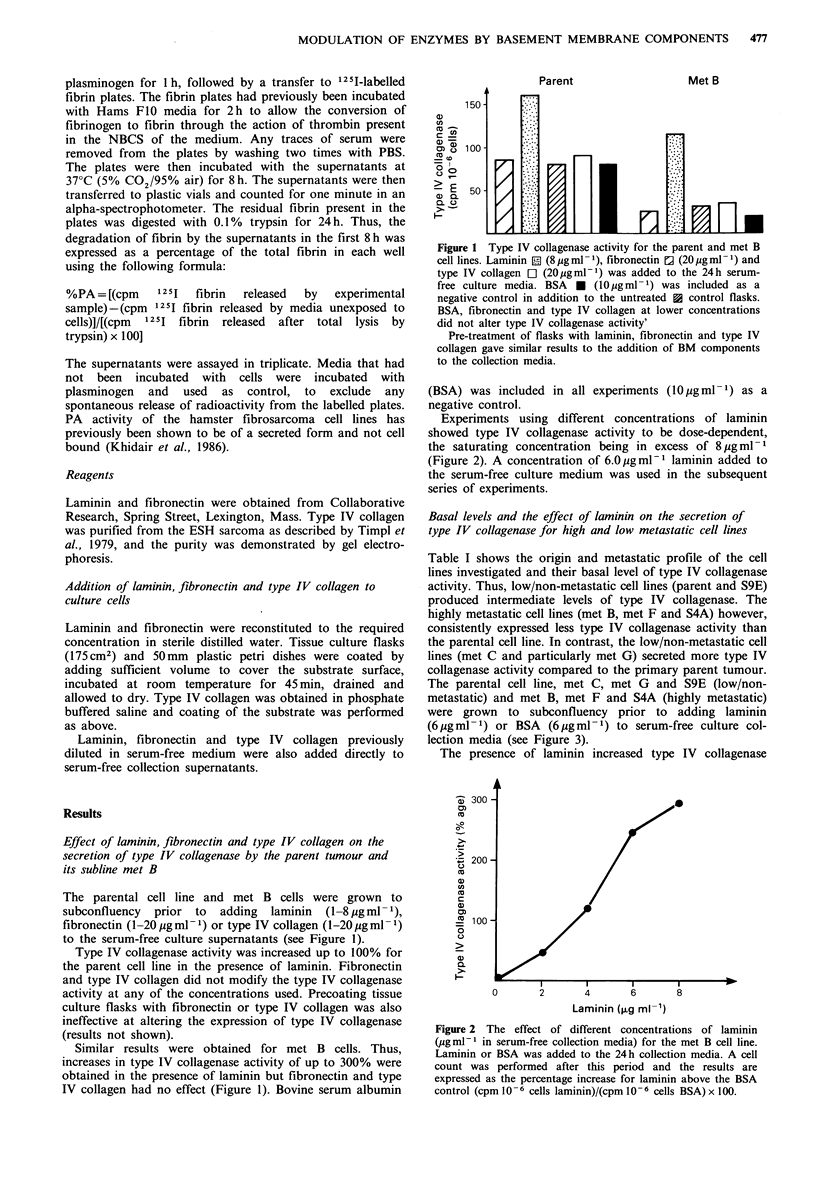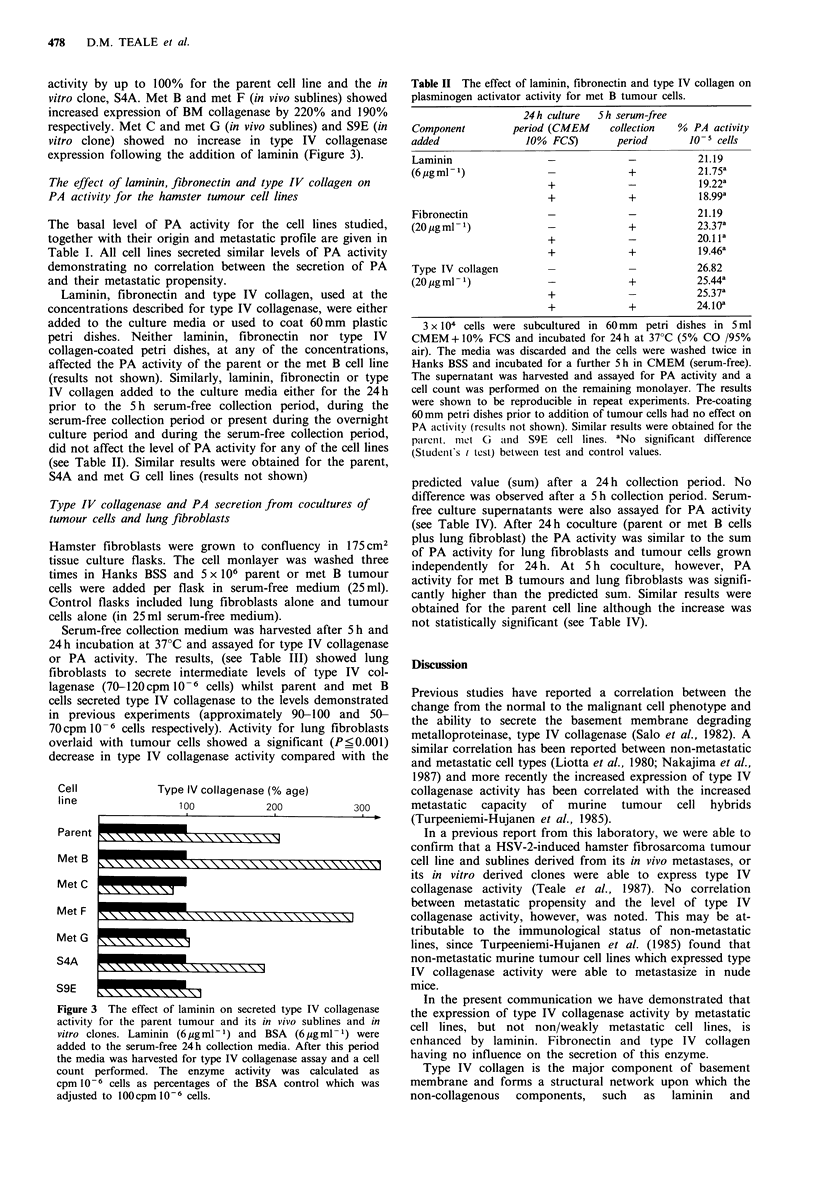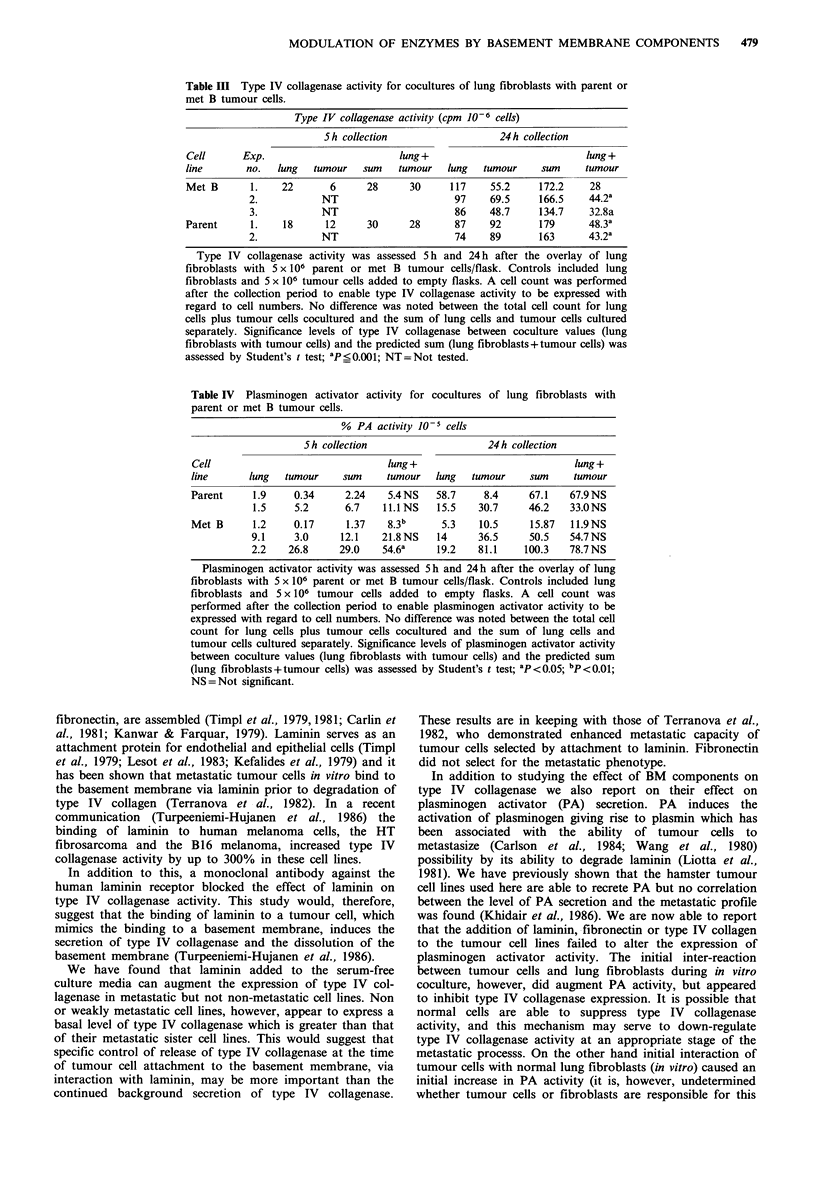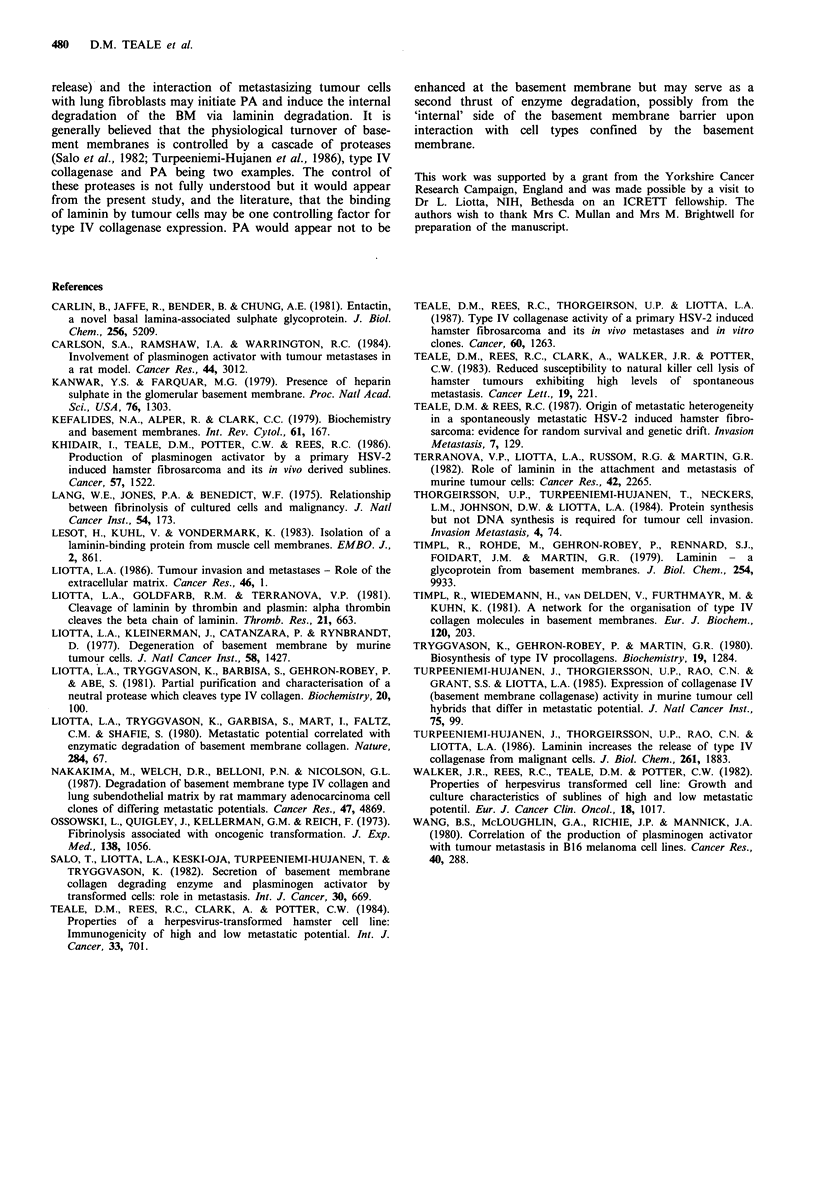# Modulation of type IV collagenase and plasminogen activator in a hamster fibrosarcoma by basement membrane components and lung fibroblasts.

**DOI:** 10.1038/bjc.1988.110

**Published:** 1988-05

**Authors:** D. M. Teale, I. A. Khidair, C. W. Potter, R. C. Rees

**Affiliations:** Department of Virology, University of Sheffield Medical School, UK.

## Abstract

The effect of basement membrane components (laminin, fibronectin and type IV collagen) and lung fibroblasts on type IV collagenase and plasminogen activator activity was investigated in a primary HSV-2-induced hamster fibrosarcoma, and its in vivo derived sublines and in vitro derived clones of varying metastatic potential. Fibronectin and type IV collagen were ineffective at influencing the expression of either type IV collagenase or plasminogen activator activity. Laminin, however, at concentrations of 1-10 micrograms ml-1 added to the serum-free culture supernatants, increased the release of type IV collagenase by up to 100% for the parental cell line. Three highly metastatic sublines (two from in vivo origin and one from in vitro cloning) showed increases of up to 300%. Non-metastatic sublines (two from in vivo origin and one from in vitro cloning), however, showed no increase in type IV collagenase activity. Plasminogen activator release from either the parental line cell or its metastatic sublines and clones, was unaffected by the addition of laminin. Addition of tumour cells to lung fibroblast monolayers resulted in an increased expression of PA activity in the supernatant, whilst type IV collagenase activity was reduced.


*Br. J. Cancer* (1988), **57**, 475–480 © The Macmillan Press Ltd., 1988

### Modulation of type IV collagenase and plasminogen activator in a hamster fibrosarcoma by basement membrane components and lung fibroblasts

D.M. Teale*, I.A. Khidair, C.W. Potter & R.C. Rees

*Department of Virology, University of Sheffield Medical School, Beech Hill Road, Sheffield S10 2RX, UK.*

**Summary** The effect of basement membrane components (laminin, fibronectin and type IV collagen) and lung fibroblasts on type IV collagenase and plasminogen activator activity was investigated in a primary HSV-2-induced hamster fibrosarcoma, and its *in vivo* derived sublines and *in vitro* derived clones of varying metastatic potential.

Fibronectin and type IV collagen were ineffective at influencing the expression of either type IV collagenase or plasminogen activator activity. Laminn, however, at concentrations of 1–10 $\mu g\,ml^{-1}$ added to the serum-free culture supernatants, increased the release of type IV collagenase by up to 100% for the parental cell line. Three highly metastatic sublines (two from *in vivo* origin and one from *in vitro* cloning) showed increases of up to 300%. Non-metastic sublines (two from *in vivo* origin and one from *in vitro* cloning), however, showed no increase in type IV collagenase activity. Plasminogen activator release from either the parental line cell or its metastatic sublines and clones, was unaffected by the addition of laminin.

Addition of tumour cells to lung fibroblast monolayers resulted in an increased expression of PA activity in the supernatant, whilst type IV collagenase activity was reduced.

Metastasizing tumour cells traverse epithelial and endothelial basement membranes (BM) whilst extravasating from the circulation and invading the target organ (Thorgeirsson *et al.*, 1984). Type IV collagenase, a metalloproteinase, has been demonstrated to be the specific enzyme capable of degrading type IV collagen, the structural back bone of the BM (Timpl *et al.*, 1981; Liotta *et al.*, 1980). Other components of the BM are non-collagenous glycoproteins such as laminin and fibronectin (Timpl *et al.*, 1979; Carlin *et al.*, 1981; Kanwar *et al.*, 1979).

Several tumours have previously been demonstrated to secrete increased quantities of type IV collagenase compared with their non-malignant cell type (Salo *et al.*, 1982). The metastatic potential of tumour cells has also been shown to correlate with their *in vitro* ability to degrade type IV collagen (Liotta *et al.*, 1980, 1981; Nakajima *et al.*, 1987) and more recently the increased expression of type IV collagenase activity has been associated with the increased metastatic activity of murine tumour cell hybrids (Turpeeniemi-Hujanen *et al.*, 1985).

In a recent report from this laboratory, we were unable to correlate type IV-collagen-degrading metalloproteinase activity of a spontaneously metastatic HSV-2 induced hamster fibrosarcoma with metastatic potential, Thus, sublines established from secondary lung nodules occurring following resection of the primary tumour, and *in vitro* derived clones of the parental tumour, of established metastatic potential, were heterogenous with respect to type IV collagenase activity (Teale *et al.*, 1987). Attachment to the basement membrane is a prerequisite to dissolution of the extracellular matrix (Liotta *et al.*, 1977, 1986) and experiments performed *in vitro* have demonstrated tumour cell binding to the basement membrane using laminin as the attachment factor (Terranova *et al.*, 1982). We have, therefore, assessed the influence of BM components, and lung fibroblasts, on the secretion of type IV collagenase in sublines and clone of defined metastatic ability in the hamster tumour model (Walker *et al.*, 1982; Teale & Rees, 1987; Teale *et al.*, 1983, 1984; Khidair *et al.*, 1986). The secretion of plasminogen activator (PA), a serine protease capable of degrading serum plasminogen converting it to plasmin, which in turn is capable of degrading fibrin and laminin (Carlson *et al.*, 1984; Liotta *et al.*, 1981), was also investigated in this system.

### Materials and methods

### *Animals*

Male, Syrian golden hamsters, aged between 6 and 10 weeks and weighing 60–90 grams were used in all experiments. The animals were obtained from a closed randomly bred colony at the University of Sheffield and have previously been shown to be syngeneic by skin grafting experiments (Potter & Jennings, unpublished) and mixed lymphocyte reactions *in vitro* (Teale, unpublished).

### *Tumours*

The HSV-2-333-2-26 cell line (parent) used in the current study was originally obtained by *in vitro* transformation of hamster embryo fibroblasts with inactivated HSV-2; this cell line was provided by Dr F. Rapp (Department of Microbiology, Pennsylvania State University, Hershey, USA).

The four sublines met B, met C, met F and met G were derived from lung nodules in hamsters whose primary parent load had previously been resected; following *in vivo* passage, *in vitro* cultures were established and used within 10 passages. Clones S4A and S9E were obtained following double cloning of the parental cell line by the limiting dilution method (Teale & Rees, 1987). Some of the characteristics of the *in vivo* and *in vitro* derived cell lines have been reported previously (Walker *et al.*, 1982; Teale & Rees, 1987; Teale *et al.*, 1983, 1984; Khidair *et al.*, 1986) and are summarised in Table I.

### *Lung fibroblasts*

Lungs from one-week old baby hamsters were minced with forceps and scalpel, digested with 0.25% trypsin (w/v) containing DNAase (0.02% w/v) and the cell suspensions seeded into 25 $cm^3$ plastic tissue culture flasks in Complete Modified Eagles Medium (CMEM) supplemented with 10% foetal calf serum (FCS), and 50 $\mu g\,ml^{-1}$ penicillin, 50 $\mu g\,ml^{-1}$ streptomycin and 50 $\mu g\,ml^{-1}$ gentamycin.

*Present address: ICI plc, Pharmaceuticals Division, Mereside, Alderley Park, Macclesfield, Cheshire SK10 4TG, UK.
Correspondence: D.M. Teale, at his present address.
Received 8 September 1987; and in revised form 18 February 1988.

**Table I** Type IV collagenase and plasminogen activator activity for the parent cell line and its *in vivo* sublines and *in vitro* clones.

| *Cell line* | *Origin* | *Metastatic ability* | *Type IV collagenase (cpm $10^{-6}$ cells)* | *% PA activity $10^{-5}$ cells* |
|---|---|---|---|---|
| Parent | primary tumour (HSV-2 transformed) fibroblasts | Low | $80 \pm 7.2$ | $20 \pm 1.1$ |
| Met B | Lung nodule | High | $40 \pm 4.3$ | $20 \pm 0.9$ |
| Met C | following | Low/none | $110 \pm 14.2$ | $29 \pm 0.6$ |
| Met F | parent tumour | High | $50 \pm 13.9$ | $36 \pm 0.9$ |
| Met G | resection | Low/none | $280 \pm 38.3$ | $30 \pm 1.3$ |
| S4A | Limiting | High | $48 \pm 9.4$ | $28 \pm 0.7$ |
| S9E | dilution clones | None | $70 \pm 6.3$ | $32 \pm 0.9$ |

The trend in results is representative of several repeat experiments. The parent tumour was originally obtained by transformation of hamster embryo fibroblasts with inactivated HSV-2. Lung nodules were obtained following primary parent tumour resection and implanted s.c. and passaged *in vivo* prior to establishment of *in vitro* cell lines. S4A and S9E were obtained following double limiting dilution cloning of the parent cell line. Metastatic ability was assessed following s.c. inoculation of $10^4$ tumour cells and the resulting tumour mass excised at 10–15 mm mean diameter. Animals were observed for signs of illness or respiratory distress and were sacrificed when moribund. Metastases occurred in the lungs, pleural cavity, kidneys and regional lymph nodes (Teale *et al.*, 1984; Teale and Rees, 1987).

*Cell cultures*

All cell lines were maintained in CMEM, and incubated at 37°C in a humidified atmosphere containing 5% $C0_2$.

*Collection of media and preparation for enzyme assay*

Four T175 $cm^2$ sub-confluent flasks of each cell line were washed X3 with Hanks balanced salt solution (HBSS) and incubated in 25 ml of serum-free CMEM for 24 hrs. The media was collected, centrifuged to remove free cells and the supernatant concentrated 100-fold with ammonium sulphate precipitation (0–60%) and dialysed against 0.2 M NaCl, 0.05 M Tris HCl, 5 mM $CaCl_2$ pH7.6 at 4°C. This preparation (1.0 ml) was stored at −20°C prior to assay for type IV collagenase.

*Preparation of substrates*

Type IV collagen was biosynthetically labelled with $^{14}$C-proline (5 mCi $mmol^{-1}$) from organ cultures of ESH sarcoma as described previously (Tryggvason *et al.*, 1980) and purified according to Liotta *et al.* (1981). Substrate preparation was checked for purity by SDS-PAGE and stored frozen in 0.5 M acetic acid until use.

*Type IV collagenase assay*

BM collagenase activity was assayed as described previously (Liotta *et al.*, 1981; Turpeeniemi-Hujanen *et al.*, 1985; Teale *et al.*, 1987) by using soluble $^3$H-proline-labelled type IV procollagen. The substrate, 6000 cpm, was added in 50 $\mu$l of reaction buffer (specific activity of 240 cpm $\mu g^{-1}$ protein). The enzyme sample was activated with 10 $\mu g\,ml^{-1}$ of trypsin for 10 min at 37°C and assayed in the presence of 50 $\mu g\,ml^{-1}$ SBTl for 10 h at 37°C. These conditions have previously been demonstrated to activate latent hamster fibrosarcoma type IV collagenase (Teale *et al.*, 1987). The reaction was terminated by inactivating the samples at 4°C for 90 min in the presence of 20 $\mu$l bovine serum albumin (5 mg $ml^{-1}$), 0.6% trichloroacetic acid (TCA) and 0.03% tannic acid. The undigested TCA precipitated substrate was centrifuged and the radioactivity of the supernatant counted in a $\beta$-scintillation counter. The amount of degraded type IV collagen was then calculated from the total amount of radio-labelled substrate (8000 cpm). Bacterial collagenase was used as a positive control to achieve maximum degradation levels whilst samples without enzyme or with EDTA or STB1 alone were included as negative controls.

Experiments to characterise the enzyme activity by SDS polyacrylamide gel electrophoresis indicated the Type IV pro-collagen substrate to yield two sets of cleavage products (results not shown) consistent with cleavage products reported in other systems (Liotta *et al.*, 1981; Teale *et al.*, 1987).

Cell counts, using a haemocytometer, were performed on trypsinised cell monolayers plus the centrifuged pellet of the collection media.

*Assay for plasminogen activator*
*Preparation of $^{125}$I-labelled fibrin plates*

Bovine fibrinogen (Sigma F8630) was purified from contaminating traces of plasminogen by the precipitation method and subsequently radiolabelled with $^{125}$I. Two hundred ml of 100 $\mu g\,ml^{-1}$ purified fibrinogen solution was mixed with the content of one vial of $^{125}$I human fibrinogen (110 $\mu$Ci) that had been resuspended in phosphate-buffered saline (PBS). Two hundred $\mu$l of the radiolabelled fibrinogen solution was added to each well of a 24-well tissue culture plate (Falcon 3047), dried at 37°C and exposed to ultraviolet (UV) light for 30 min to minimize the risk of contamination. Plates were stored at −20°C until required for use.

*Preparation of plasminogen*

Plasminogen was extracted from rabbit serum by affinity chromatography with lysine sepharose 4B as previously described. Plasminogen was titrated by the addition of increasing quantities of plasminogen to a constant amount of supernatant from the cell culture. The amount of plasminogen that gave maximum release of radioactivity was determined and used in all subsequent experiments (100 $\mu$l).

*Extracellular PA activity*

*In vitro* cell cultures were prepared from *in vivo* tissue (normal or tumour) as previously described. The cells were cultured in 60-mm plastic petri dishes (Sterilin, 303) in 5 ml of Hams F10 media (10% NBCS) at a concentration of $3 \times 10^4$ cells $ml^{-1}$ and incubated for 24 h at 37°C (5% CO /95% air). The cells were washed twice with PBS to remove traces of serum and incubated with 5 ml Hams F10 media (serum-free) for an additional 5 h. The supernatants were centrifuged at 200 rpm for 5 min, collected, and stored at −20°C. The remaining cells were treated with trypsin and counted so that activities could be expressed with respect to the cell number. The fibrinolytic assay was performed by incubating 400 $\mu$l of the supernatants with 100 $\mu$l of plasminogen for 1 h, followed by a transfer to $^{125}$I-labelled fibrin plates. The fibrin plates had previously been incubated with Hams F10 media for 2 h to allow the conversion of fibrinogen to fibrin through the action of thrombin present in the NBCS of the medium. Any traces of serum were removed from the plates by washing two times with PBS. The plates were then incubated with the supernatants at 37°C (5% $CO_2$/95% air) for 8 h. The supernatants were then transferred to plastic vials and counted for one minute in an alpha-spectrophotometer. The residual fibrin present in the plates was digested with 0.1% trypsin for 24 h. Thus, the degradation of fibrin by the supernatants in the first 8 h was expressed as a percentage of the total fibrin in each well using the following formula:

%PA = [(cpm $^{125}$I fibrin released by experimental sample) − (cpm $^{125}$I fibrin released by media unexposed to cells)]/[(cpm $^{125}$I fibrin released after total lysis by trypsin) × 100]

The supernatants were assayed in triplicate. Media that had not been incubated with cells were incubated with plasminogen and used as control, to exclude any spontaneous release of radioactivity from the labelled plates. PA activity of the hamster fibrosarcoma cell lines has previously been shown to be of a secreted form and not cell bound (Khidair *et al.*, 1986).

*Reagents*

Laminin and fibronectin were obtained from Collaborative Research, Spring Street, Lexington, Mass. Type IV collagen was purified from the ESH sarcoma as described by Timpl *et al.*, 1979, and the purity was demonstrated by gel electrophoresis.

*Addition of laminin, fibronectin and type IV collagen to culture cells*

Laminin and fibronectin were reconstituted to the required concentration in sterile distilled water. Tissue culture flasks (175 cm$^2$) and 50 mm plastic petri dishes were coated by adding sufficient volume to cover the substrate surface, incubated at room temperature for 45 min, drained and allowed to dry. Type IV collagen was obtained in phosphate buffered saline and coating of the substrate was performed as above.

Laminin, fibronectin and type IV collagen previously diluted in serum-free medium were also added directly to serum-free collection supernatants.

### Results

*Effect of laminin, fibronectin and type IV collagen on the secretion of type IV collagenase by the parent tumour and its subline met B*

The parental cell line and met B cells were grown to subconfluency prior to adding laminin (1–8 μg ml$^{-1}$), fibronectin (1–20 μg ml$^{-1}$) or type IV collagen (1–20 μg ml$^{-1}$) to the serum-free culture supernatants (see Figure 1).

Type IV collagenase activity was increased up to 100% for the parent cell line in the presence of laminin. Fibronectin and type IV collagen did not modify the type IV collagenase activity at any of the concentrations used. Precoating tissue culture flasks with fibronectin or type IV collagen was also ineffective at altering the expression of type IV collagenase (results not shown).

Similar results were obtained for met B cells. Thus, increases in type IV collagenase activity of up to 300% were obtained in the presence of laminin but fibronectin and type IV collagen had no effect (Figure 1). Bovine serum albumin

**Figure 1** Type IV collagenase activity for the parent and met B cell lines. Laminin (8 μg ml$^{-1}$), fibronectin (20 μg ml$^{-1}$) and type IV collagen (20 μg ml$^{-1}$) was added to the 24 h serum-free culture media. BSA (10 μg ml$^{-1}$) was included as a negative control in addition to the untreated control flasks. BSA, fibronectin and type IV collagen at lower concentrations did not alter type IV collagenase activity'

Pre-treatment of flasks with laminin, fibronectin and type IV collagen gave similar results to the addition of BM components to the collection media.

(BSA) was included in all experiments (10 μg ml$^{-1}$) as a negative control.

Experiments using different concentrations of laminin showed type IV collagenase activity to be dose-dependent, the saturating concentration being in excess of 8 μg ml$^{-1}$ (Figure 2). A concentration of 6.0 μg ml$^{-1}$ laminin added to the serum-free culture medium was used in the subsequent series of experiments.

*Basal levels and the effect of laminin on the secretion of type IV collagenase for high and low metastatic cell lines*

Table I shows the origin and metastatic profile of the cell lines investigated and their basal level of type IV collagenase activity. Thus, low/non-metastatic cell lines (parent and S9E) produced intermediate levels of type IV collagenase. The highly metastatic cell lines (met B, met F and S4A) however, consistently expressed less type IV collagenase activity than the parental cell line. In contrast, the low/non-metastatic cell lines (met C and particularly met G) secreted more type IV collagenase activity compared to the primary parent tumour. The parental cell line, met C, met G and S9E (low/non-metastatic) and met B, met F and S4A (highly metastatic) were grown to subconfluency prior to adding laminin (6 μg ml$^{-1}$) or BSA (6 μg ml$^{-1}$) to serum-free culture collection media (see Figure 3).

The presence of laminin increased type IV collagenase

**Figure 2** The effect of different concentrations of laminin (μg ml$^{-1}$ in serum-free collection media) for the met B cell line. Laminin or BSA was added to the 24 h collection media. A cell count was performed after this period and the results are expressed as the percentage increase for laminin above the BSA control (cpm 10$^{-6}$ cells laminin)/(cpm 10$^{-6}$ cells BSA) × 100.

activity by up to 100% for the parent cell line and the *in vitro* clone, S4A. Met B and met F (*in vivo* sublines) showed increased expression of BM collagenase by 220% and 190% respectively. Met C and met G (*in vivo* sublines) and S9E (*in vitro* clone) showed no increase in type IV collagenase expression following the addition of laminin (Figure 3).

### *The effect of laminin, fibronectin and type IV collagen on PA activity for the hamster tumour cell lines*

The basal level of PA activity for the cell lines studied, together with their origin and metastatic profile are given in Table I. All cell lines secreted similar levels of PA activity demonstrating no correlation between the secretion of PA and their metastatic propensity.

Laminin, fibronectin and type IV collagen, used at the concentrations described for type IV collagenase, were either added to the culture media or used to coat 60 mm plastic petri dishes. Neither laminin, fibronectin nor type IV collagen-coated petri dishes, at any of the concentrations, affected the PA activity of the parent or the met B cell line (results not shown). Similarly, laminin, fibronectin or type IV collagen added to the culture media either for the 24 h prior to the 5 h serum-free collection period, during the serum-free collection period or present during the overnight culture period and during the serum-free collection period, did not affect the level of PA activity for any of the cell lines (see Table II). Similar results were obtained for the parent, S4A and met G cell lines (results not shown)

### *Type IV collagenase and PA secretion from cocultures of tumour cells and lung fibroblasts*

Hamster fibroblasts were grown to confluency in 175 $cm^2$ tissue culture flasks. The cell monlayer was washed three times in Hanks BSS and $5 \times 10^6$ parent or met B tumour cells were added per flask in serum-free medium (25 ml). Control flasks included lung fibroblasts alone and tumour cells alone (in 25 ml serum-free medium).

Serum-free collection medium was harvested after 5 h and 24 h incubation at 37°C and assayed for type IV collagenase or PA activity. The results, (see Table III) showed lung fibroblasts to secrete intermediate levels of type IV collagenase (70–120 cpm $10^{-6}$ cells) whilst parent and met B cells secreted type IV collagenase to the levels demonstrated in previous experiments (approximately 90–100 and 50–70 cpm $10^{-6}$ cells respectively). Activity for lung fibroblasts overlaid with tumour cells showed a significant ($P \leq 0.001$) decrease in type IV collagenase activity compared with the

**Figure 3** The effect of laminin on secreted type IV collagenase activity for the parent tumour and its *in vivo* sublines and *in vitro* clones. Laminin (6 $\mu g\,ml^{-1}$) and BSA (6 $\mu g\,ml^{-1}$) were added to the serum-free 24 h collection media. After this period the media was harvested for type IV collagenase assay and a cell count performed. The enzyme activity was calculated as cpm $10^{-6}$ cells as percentages of the BSA control which was adjusted to 100 cpm $10^{-6}$ cells.

**Table II** The effect of laminin, fibronectin and type IV collagen on plasminogen activator activity for met B tumour cells.

| *Component added* | *24 h culture period (CMEM 10% FCS)* | *5 h serum-free collection period* | *% PA activity $10^{-5}$ cells* |
|---|---|---|---|
| Laminin | – | – | 21.19 |
| (6 $\mu g\,ml^{-1}$) | – | + | 21.75[a] |
| | + | – | 19.22[a] |
| | + | + | 18.99[a] |
| Fibronectin | – | – | 21.19 |
| (20 $\mu g\,ml^{-1}$) | – | + | 23.37[a] |
| | + | – | 20.11[a] |
| | + | + | 19.46[a] |
| Type IV collagen | – | – | 26.82 |
| (20 $\mu g\,ml^{-1}$) | – | + | 25.44[a] |
| | + | – | 25.37[a] |
| | + | + | 24.10[a] |

$3 \times 10^4$ cells were subcultured in 60 mm petri dishes in 5 ml CMEM + 10% FCS and incubated for 24 h at 37°C (5% CO /95% air). The media was discarded and the cells were washed twice in Hanks BSS and incubated for a further 5 h in CMEM (serum-free). The supernatant was harvested and assayed for PA activity and a cell count was performed on the remaining monolayer. The results were shown to be reproducible in repeat experiments. Pre-coating 60 mm petri dishes prior to addition of tumour cells had no effect on PA activity (results not shown). Similar results were obtained for the parent, met G and S9E cell lines. [a]No significant difference (Student's *t* test) between test and control values.

predicted value (sum) after a 24 h collection period. No difference was observed after a 5 h collection period. Serum-free culture supernatants were also assayed for PA activity (see Table IV). After 24 h coculture (parent or met B cells plus lung fibroblast) the PA activity was similar to the sum of PA activity for lung fibroblasts and tumour cells grown independently for 24 h. At 5 h coculture, however, PA activity for met B tumours and lung fibroblasts was significantly higher than the predicted sum. Similar results were obtained for the parent cell line although the increase was not statistically significant (see Table IV).

### Discussion

Previous studies have reported a correlation between the change from the normal to the malignant cell phenotype and the ability to secrete the basement membrane degrading metalloproteinase, type IV collagenase (Salo *et al.*, 1982). A similar correlation has been reported between non-metastatic and metastatic cell types (Liotta *et al.*, 1980; Nakajima *et al.*, 1987) and more recently the increased expression of type IV collagenase activity has been correlated with the increased metastatic capacity of murine tumour cell hybrids (Turpeeniemi-Hujanen *et al.*, 1985).

In a previous report from this laboratory, we were able to confirm that a HSV-2-induced hamster fibrosarcoma tumour cell line and sublines derived from its *in vivo* metastases, or its *in vitro* derived clones were able to express type IV collagenase activity (Teale *et al.*, 1987). No correlation between metastatic propensity and the level of type IV collagenase activity, however, was noted. This may be attributable to the immunological status of non-metastatic lines, since Turpeeniemi-Hujanen *et al.* (1985) found that non-metastatic murine tumour cell lines which expressed type IV collagenase activity were able to metastasize in nude mice.

In the present communication we have demonstrated that the expression of type IV collagenase activity by metastatic cell lines, but not non/weakly metastatic cell lines, is enhanced by laminin. Fibronectin and type IV collagen having no influence on the secretion of this enzyme.

Type IV collagen is the major component of basement membrane and forms a structural network upon which the non-collagenous components, such as laminin and

**Table III** Type IV collagenase activity for cocultures of lung fibroblasts with parent or met B tumour cells.

| | | *Type IV collagenase activity (cpm $10^{-6}$ cells)* | | | | | | | |
|---|---|---|---|---|---|---|---|---|---|
| | | *5 h collection* | | | | *24 h collection* | | | |
| *Cell line* | *Exp. no.* | *lung* | *tumour* | *sum* | *lung + tumour* | *lung* | *tumour* | *sum* | *lung + tumour* |
| Met B | 1. | 22 | 6 | 28 | 30 | 117 | 55.2 | 172.2 | 28 |
| | 2. | | NT | | | 97 | 69.5 | 166.5 | 44.2[a] |
| | 3. | | NT | | | 86 | 48.7 | 134.7 | 32.8a |
| Parent | 1. | 18 | 12 | 30 | 28 | 87 | 92 | 179 | 48.3[a] |
| | 2. | | NT | | | 74 | 89 | 163 | 43.2[a] |

Type IV collagenase activity was assessed 5 h and 24 h after the overlay of lung fibroblasts with $5\times10^6$ parent or met B tumour cells/flask. Controls included lung fibroblasts and $5\times10^6$ tumour cells added to empty flasks. A cell count was performed after the collection period to enable type IV collagenase activity to be expressed with regard to cell numbers. No difference was noted between the total cell count for lung cells plus tumour cells cocultured and the sum of lung cells and tumour cells cultured separately. Significance levels of type IV collagenase between coculture values (lung fibroblasts with tumour cells) and the predicted sum (lung fibroblasts + tumour cells) was assessed by Student's $t$ test; [a]$P \leq 0.001$; NT = Not tested.

**Table IV** Plasminogen activator activity for cocultures of lung fibroblasts with parent or met B tumour cells.

| | *% PA activity $10^{-5}$ cells* | | | | | | | |
|---|---|---|---|---|---|---|---|---|
| | *5 h collection* | | | | *24 h collection* | | | |
| *Cell line* | *lung* | *tumour* | *sum* | *lung + tumour* | *lung* | *tumour* | *sum* | *lung + tumour* |
| Parent | 1.9 | 0.34 | 2.24 | 5.4 NS | 58.7 | 8.4 | 67.1 | 67.9 NS |
| | 1.5 | 5.2 | 6.7 | 11.1 NS | 15.5 | 30.7 | 46.2 | 33.0 NS |
| Met B | 1.2 | 0.17 | 1.37 | 8.3[b] | 5.3 | 10.5 | 15.87 | 11.9 NS |
| | 9.1 | 3.0 | 12.1 | 21.8 NS | 14 | 36.5 | 50.5 | 54.7 NS |
| | 2.2 | 26.8 | 29.0 | 54.6[a] | 19.2 | 81.1 | 100.3 | 78.7 NS |

Plasminogen activator activity was assessed 5 h and 24 h after the overlay of lung fibroblasts with $5\times10^6$ parent or met B tumour cells/flask. Controls included lung fibroblasts and $5\times10^6$ tumour cells added to empty flasks. A cell count was performed after the collection period to enable plasminogen activator activity to be expressed with regard to cell numbers. No difference was noted between the total cell count for lung cells plus tumour cells cocultured and the sum of lung cells and tumour cells cultured separately. Significance levels of plasminogen activator activity between coculture values (lung fibroblasts with tumour cells) and the predicted sum (lung fibroblasts + tumour cells) was assessed by Student's $t$ test; [a]$P<0.05$; [b]$P<0.01$; NS = Not significant.

fibronectin, are assembled (Timpl *et al.*, 1979, 1981; Carlin *et al.*, 1981; Kanwar & Farquar, 1979). Laminin serves as an attachment protein for endothelial and epithelial cells (Timpl *et al.*, 1979; Lesot *et al.*, 1983; Kefalides *et al.*, 1979) and it has been shown that metastatic tumour cells *in vitro* bind to the basement membrane via laminin prior to degradation of type IV collagen (Terranova *et al.*, 1982). In a recent communication (Turpeeniemi-Hujanen *et al.*, 1986) the binding of laminin to human melanoma cells, the HT fibrosarcoma and the B16 melanoma, increased type IV collagenase activity by up to 300% in these cell lines.

In addition to this, a monoclonal antibody against the human laminin receptor blocked the effect of laminin on type IV collagenase activity. This study would, therefore, suggest that the binding of laminin to a tumour cell, which mimics the binding to a basement membrane, induces the secretion of type IV collagenase and the dissolution of the basement membrane (Turpeeniemi-Hujanen *et al.*, 1986).

We have found that laminin added to the serum-free culture media can augment the expression of type IV collagenase in metastatic but not non-metastatic cell lines. Non or weakly metastatic cell lines, however, appear to express a basal level of type IV collagenase which is greater than that of their metastatic sister cell lines. This would suggest that specific control of release of type IV collagenase at the time of tumour cell attachment to the basement membrane, via interaction with laminin, may be more important than the continued background secretion of type IV collagenase. These results are in keeping with those of Terranova *et al.*, 1982, who demonstrated enhanced metastatic capacity of tumour cells selected by attachment to laminin. Fibronectin did not select for the metastatic phenotype.

In addition to studying the effect of BM components on type IV collagenase we also report on their effect on plasminogen activator (PA) secretion. PA induces the activation of plasminogen giving rise to plasmin which has been associated with the ability of tumour cells to metastasize (Carlson *et al.*, 1984; Wang *et al.*, 1980) possibility by its ability to degrade laminin (Liotta *et al.*, 1981). We have previously shown that the hamster tumour cell lines used here are able to recrete PA but no correlation between the level of PA secretion and the metastatic profile was found (Khidair *et al.*, 1986). We are now able to report that the addition of laminin, fibronectin or type IV collagen to the tumour cell lines failed to alter the expression of plasminogen activator activity. The initial inter-reaction between tumour cells and lung fibroblasts during *in vitro* coculture, however, did augment PA activity, but appeared to inhibit type IV collagenase expression. It is possible that normal cells are able to suppress type IV collagenase activity, and this mechanism may serve to down-regulate type IV collagenase activity at an appropriate stage of the metastatic processs. On the other hand initial interaction of tumour cells with normal lung fibroblasts (*in vitro*) caused an initial increase in PA activity (it is, however, undetermined whether tumour cells or fibroblasts are responsible for this release) and the interaction of metastasizing tumour cells with lung fibroblasts may initiate PA and induce the internal degradation of the BM via laminin degradation. It is generally believed that the physiological turnover of basement membranes is controlled by a cascade of proteases (Salo *et al.*, 1982; Turpeeniemi-Hujanen *et al.*, 1986), type IV collagenase and PA being two examples. The control of these proteases is not fully understood but it would appear from the present study, and the literature, that the binding of laminin by tumour cells may be one controlling factor for type IV collagenase expression. PA would appear not to be enhanced at the basement membrane but may serve as a second thrust of enzyme degradation, possibly from the 'internal' side of the basement membrane barrier upon interaction with cell types confined by the basement membrane.

This work was supported by a grant from the Yorkshire Cancer Research Campaign, England and was made possible by a visit to Dr L. Liotta, NIH, Bethesda on an ICRETT fellowship. The authors wish to thank Mrs C. Mullan and Mrs M. Brightwell for preparation of the manuscript.

### References

CARLIN, B., JAFFE, R., BENDER, B. & CHUNG, A.E. (1981). Entactin, a novel basal lamina-associated sulphate glycoprotein. *J. Biol. Chem.*, **256**, 5209.

CARLSON, S.A., RAMSHAW, I.A. & WARRINGTON, R.C. (1984). Involvement of plasminogen activator with tumour metastases in a rat model. *Cancer Res.*, **44**, 3012.

KANWAR, Y.S. & FARQUAR, M.G. (1979). Presence of heparin sulphate in the glomerular basement membrane. *Proc. Natl Acad. Sci., USA*, **76**, 1303.

KEFALIDES, N.A., ALPER, R. & CLARK, C.C. (1979). Biochemistry and basement membranes. *Int. Rev. Cytol.*, **61**, 167.

KHIDAIR, I., TEALE, D.M., POTTER, C.W. & REES, R.C. (1986). Production of plasminogen activator by a primary HSV-2 induced hamster fibrosarcoma and its *in vivo* derived sublines. *Cancer*, **57**, 1522.

LANG, W.E., JONES, P.A. & BENEDICT, W.F. (1975). Relationship between fibrinolysis of cultured cells and malignancy. *J. Natl Cancer Inst.*, **54**, 173.

LESOT, H., KUHL, V. & VONDERMARK, K. (1983). Isolation of a laminin-binding protein from muscle cell membranes. *EMBO. J.*, **2**, 861.

LIOTTA, L.A. (1986). Tumour invasion and metastases – Role of the extracellular matrix. *Cancer Res.*, **46**, 1.

LIOTTA, L.A., GOLDFARB, R.M. & TERRANOVA, V.P. (1981). Cleavage of laminin by thrombin and plasmin: alpha thrombin cleaves the beta chain of laminin. *Thromb. Res.*, **21**, 663.

LIOTTA, L.A., KLEINERMAN, J., CATANZARA, P. & RYNBRANDT, D. (1977). Degeneration of basement membrane by murine tumour cells. *J. Natl Cancer Inst.*, **58**, 1427.

LIOTTA, L.A., TRYGGVASON, K., BARBISA, S., GEHRON-ROBEY, P. & ABE, S. (1981). Partial purification and characterisation of a neutral protease which cleaves type IV collagen. *Biochemistry*, **20**, 100.

LIOTTA, L.A., TRYGGVASON, K., GARBISA, S., MART, I., FALTZ, C.M. & SHAFIE, S. (1980). Metastatic potential correlated with enzymatic degradation of basement membrane collagen. *Nature*, **284**, 67.

NAKAKIMA, M., WELCH, D.R., BELLONI, P.N. & NICOLSON, G.L. (1987). Degradation of basement membrane type IV collagen and lung subendothelial matrix by rat mammary adenocarcinoma cell clones of differing metastatic potentials. *Cancer Res.*, **47**, 4869.

OSSOWSKI, L., QUIGLEY, J., KELLERMAN, G.M. & REICH, F. (1973). Fibrinolysis associated with oncogenic transformation. *J. Exp. Med.*, **138**, 1056.

SALO, T., LIOTTA, L.A., KESKI-OJA, TURPEENIEMI-HUJANEN, T. & TRYGGVASON, K. (1982). Secretion of basement membrane collagen degrading enzyme and plasminogen activator by transformed cells: role in metastasis. *Int. J. Cancer*, **30**, 669.

TEALE, D.M., REES, R.C., CLARK, A. & POTTER, C.W. (1984). Properties of a herpesvirus-transformed hamster cell line: Immunogenicity of high and low metastatic potential. *Int. J. Cancer*, **33**, 701.

TEALE, D.M., REES, R.C., THORGEIRSON, U.P. & LIOTTA, L.A. (1987). Type IV collagenase activity of a primary HSV-2 induced hamster fibrosarcoma and its *in vivo* metastases and *in vitro* clones. *Cancer*, **60**, 1263.

TEALE, D.M., REES, R.C., CLARK, A., WALKER, J.R. & POTTER, C.W. (1983). Reduced susceptibility to natural killer cell lysis of hamster tumours exhibiting high levels of spontaneous metastasis. *Cancer Lett.*, **19**, 221.

TEALE, D.M. & REES, R.C. (1987). Origin of metastatic heterogeneity in a spontaneously metastatic HSV-2 induced hamster fibrosarcoma: evidence for random survival and genetic drift. *Invasion Metastasis*, **7**, 129.

TERRANOVA, V.P., LIOTTA, L.A., RUSSOM, R.G. & MARTIN, G.R. (1982). Role of laminin in the attachment and metastasis of murine tumour cells: *Cancer Res.*, **42**, 2265.

THORGEIRSSON, U.P., TURPEENIEMI-HUJANEN, T., NECKERS, L.M., JOHNSON, D.W. & LIOTTA, L.A. (1984). Protein synthesis but not DNA synthesis is required for tumour cell invasion. *Invasion Metastasis*, **4**, 74.

TIMPL, R., ROHDE, M., GEHRON-ROBEY, P., RENNARD, S.J., FOIDART, J.M. & MARTIN, G.R. (1979). Laminin – a glycoprotein from basement membranes. *J. Biol. Chem.*, **254**, 9933.

TIMPL, R., WIEDEMANN, H., VAN DELDEN, V., FURTHMAYR, M. & KUHN, K. (1981). A network for the organisation of type IV collagen molecules in basement membranes. *Eur. J. Biochem.*, **120**, 203.

TRYGGVASON, K., GEHRON-ROBEY, P. & MARTIN, G.R. (1980). Biosynthesis of type IV procollagens. *Biochemistry*, **19**, 1284.

TURPEENIEMI-HUJANEN, J., THORGIERSSON, U.P., RAO, C.N. & GRANT, S.S. & LIOTTA, L.A. (1985). Expression of collagenase IV (basement membrane collagenase) activity in murine tumour cell hybrids that differ in metastatic potential. *J. Natl Cancer Inst.*, **75**, 99.

TURPEENIEMI-HUJANEN, J., THORGEIRSSON, U.P., RAO, C.N. & LIOTTA, L.A. (1986). Laminin increases the release of type IV collagenase from malignant cells. *J. Biol. Chem.*, **261**, 1883.

WALKER, J.R., REES, R.C., TEALE, D.M. & POTTER, C.W. (1982). Properties of herpesvirus transformed cell line: Growth and culture characteristics of sublines of high and low metastatic potentil. *Eur. J. Cancer Clin. Oncol.*, **18**, 1017.

WANG, B.S., McLOUGHLIN, G.A., RICHIE, J.P. & MANNICK, J.A. (1980). Correlation of the production of plasminogen activator with tumour metastasis in B16 melanoma cell lines. *Cancer Res.*, **40**, 288.